# A commentary on “Sleep patterns, genetic susceptibility, and digestive diseases: a large-scale longitudinal cohort study”

**DOI:** 10.1097/JS9.0000000000002914

**Published:** 2025-07-02

**Authors:** Hua Yang, Jie Yang, Chunxiang Zhang

**Affiliations:** a Department of Medical Psych 5, Huai’an NO.3 People’s Hospital, Huai’an, Jiangsu, China; b Department of Medical Psychology, Huai’an NO.3 People’s Hospital, Huai’an, Jiangsu, China

*Dear Editor,*

We read the article in the *International Journal of Surgery* with considerable interest. Ma *et al*^[1]^ conducted a prospective observational study that aimed to explore the association between the risk of digestive diseases and a healthy sleep pattern. It offers a comprehensive assessment of a healthy sleep pattern that has been connected to a decreased incidence of digestive issues, regardless of genetic susceptibility. Future studies must, however, address a number of important concerns.

First, there’s the potential for reverse causality. The idea of reverse causality, in which disorders of the digestive system impact sleep quality instead of the other way around, cannot be completely ruled out by the study design. Although the authors excluded participants with pre-existing digestive issues at baseline and conducted lagged analyses, subclinical or undiagnosed digestive problems may still exist. For example, undetected gastroesophageal reflux disease (GERD) could lead to insomnia, which might statistically appear as a link between poor sleep and GERD. Longer lag times or prospective designs could help further reduce this bias.

Second, the outcomes measurement’s limitations. ICD-10 codes and electronic medical records are used to diagnose disorders of the digestive system, which could result in missed or incorrect diagnoses. For instance, diagnosing non-alcoholic fatty liver disease (NAFLD) typically requires imaging or biopsy confirmation, but the study did not specify the exact diagnostic methods. Moreover, certain conditions, such as irritable bowel syndrome, primarily depend on symptoms rather than objective indicators, potentially leading to diagnostic biases. The lack of stratified analysis of the severity of different diseases also limits the clinical significance of the findings.

Third, consider the challenge of calculating sleep scores. The Healthy Sleep Score categorizes five sleep behaviors into two simple categories (low risk =1 point, high risk =0 points), which may oversimplify the complexity of sleep patterns. For example, it considers “7-8 hours of sleep” as healthy, while slightly less or more than this range is deemed high-risk, overlooking individual differences^[2,3]^. While attempts have been made to improve the weighted sleep score, the sources and rationale for the weights (β coefficients) are not adequately explained. Furthermore, the consistency of the scores across different subgroups, such as age and gender, has not been verified.

In conclusion, this study offers important evidence regarding the connection between disorders of the digestive system and sleep. By including objective sleep monitoring, incorporating more varied groups, improving clinical translation research, and honing genetic risk analysis, future studies can further validate and broaden these findings. All of this was noted in relation to the TITAN guideline, which calls for transparency in the reporting of artificial intelligence^[4]^.

## Data Availability

No data was used in this Letter to the Editor.
